# A Secure and Efficient Handover Authentication Protocol for Wireless Networks

**DOI:** 10.3390/s140711379

**Published:** 2014-06-26

**Authors:** Weijia Wang, Lei Hu

**Affiliations:** 1 School of Science, Beijing Jiaotong University, Beijing 100044, China; 2 The State Key Laboratory of Information Security, Institute of Information Engineering, Chinese Academy of Sciences, Beijing 100093, China; E-Mail: hu@is.ac.cn

**Keywords:** wireless network, security, privacy, handover authentication

## Abstract

Handover authentication protocol is a promising access control technology in the fields of WLANs and mobile wireless sensor networks. In this paper, we firstly review an efficient handover authentication protocol, named PairHand, and its existing security attacks and improvements. Then, we present an improved key recovery attack by using the linearly combining method and reanalyze its feasibility on the improved PairHand protocol. Finally, we present a new handover authentication protocol, which not only achieves the same desirable efficiency features of PairHand, but enjoys the provable security in the random oracle model.

## 1. Introduction

In today's world, wireless communication networks are ubiquitous, and mobile handheld devices, such as PDAs, smart phones and laptop PCs, have a wide influence on various aspects of people's lives. To overcome the restriction of geographical coverage, seamless access services are highly desirable for WLANs and mobile wireless sensor networks (WSNs), but how to ensure the security and efficiency of this process is still challenging. Recently, as a promising seamless access control technology, handover authentication protocols have received much attention [1–12]. A handover authentication scenario is always assumed to involve three kinds of parties: mobile nodes (MNs), access points (APs) and the authentication server (AS). An MN is a registered user on AS, who accesses its subscribed services by connecting any AP. An AP acts as a guarantor for vouching for an MN as a legitimate subscriber. When an MN leaves the service area of the current AP (e.g., AP1) and tries to connect a new AP (e.g., AP2), the new AP will start its handover authentication process to identify the MN. If the authentication succeeds, a session key will be built between the MN and AP2 to escort the MN's later access. Otherwise, the requirement for accessing will be rejected by AP2. A promising application of this kind of protocol appears in three-tiered mobile WSNs [13], which consist of a base station, access points, mobile agents and sensor nodes. In the highest layer, the base station works as the AS to deploy access points and to register mobile agents by granting the corresponding authentication keys. The access points are the APs with the task of receiving and verifying the message from the medium layer. The medium layer is composed of the mobile agents, which can be mobile phones, vehicles, men and even animal, acting as the MNs and responsible for gathering data from the sensor nodes in the lowest layer and, then, forwarding to the upper layer.

Recently, He *et al.* [14] introduced an interesting handover authentication protocol, named PairHand. For improving the communication efficiency and reducing the burden on AS, PairHand only requires two handshakes between MN and AP for mutual authentication and key establishment, instead of relying on the participation of AS. Furthermore, considering the high cost and the inconvenience of revoking users due to using a group signature in the authentication process, PairHand makes its construction directly based on the pairing-based cryptography and uses a pool of shorter-lived pseudonyms to protect users' privacy. Unfortunately, shortly after, He *et al.* [15] found that there is a serious design weakness in PairHand protocol that enables an adversary to easily obtain the private key from the message transported in the first round of the protocol and presented an improvement by utilizing a composite order bilinear group, claiming that the improved version fixes the security problem without losing any of the desirable feature of PairHand. However, Yeo *et al.* [16] showed that if an attacker obtains multiple authenticated messages generated with the same pseudo-ID, it will be likely to recover the private key of the mobile node. Furthermore, Yeo *et al.* [16] and Tsai *et al.* [17] pointed out another dilemma of the improved version that suggested that the 160-bit composite is insecure, but using a 1,024-bit composite-order group will lead to a great drop in the efficiency. At the same time, Tsai *et al.* [17] presented a provably secure handover authentication protocol, which solves the above security problem, but increases the size of the public key.

In this paper, we provide a linear combination method to reduce the number of captured signatures corresponding to the same pseudo-ID required by the key recovery attack on the improved PairHand [15]. By repeatedly linearly combining arbitrary two-captured signatures from the same pseudo-ID in a random way, the attacker can compute out the private key of MN with a very high probability. To improve the security without losing the desirable features, we present a new handover authentication protocol that overcomes the security weakness of the original PairHand and achieves the same level of high efficiency. Finally, in the random oracle model, we prove that this protocol enjoys both semantic security and authentication security.

## 2. The Bilinear Maps and Complexity Implications

In this section, we briefly recall bilinear maps and some difficult problems that will be used in the followings.

Let 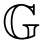 be a cyclic additive group of composite order *q* and 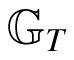 be a cyclic multiplicative group of the same order. Let *e*: 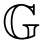 × 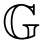 → 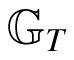 be a bilinear map that satisfies the following properties.

- Bilinearity: *e*(*aP*, *bQ*) = (*P*, *Q*)*^ab^* for ∀*P*, *Q* ∈ 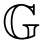 and ∀*a*, $b\in Z_{q}^{*}$
- Non-degeneracy: *e*(*P*, *P*) ≠ 1 for *P* ≠ 0.
- Computability: there exists an efficient algorithm to compute *e*(*P*, *Q*) for ∀*P*, *Q* ∈ 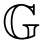.

Computational Diffie–Hellman (CDH) assumption: Given *P*, *aP* and *bP* for some *a*, $b\in Z_{q}^{*}$, it is computationally intractable to compute the value *abP*.

Bilinear Diffie–Hellman (BDH) assumption: Given *P*, *aP*, *bP* and *cP* for some *a*, *b*, $c\in Z_{q}^{*}$, it is computationally intractable to compute the value *e*(*P*, *P*)*^abc^*.

## 3. Security Model

Generally, there are two kinds of handovers: a hard handover and a soft handover. The difference between them is that in a hard handover, the former connection with AP1 is broken before the new connection is established between MN and AP2, while in a soft handover, MN can retain the connection with AP1 after building the new connection with AP2. For simplicity, it is assumed that there is no communication among APs and that handover authentication protocols perform in the hard handover model. In the following, we present the formal security model for handover authentication protocols, which follows the approach initiated by Bellare and Rogway [18,19] and modified by Bresson *et al.* [20].

### 3.1. Communication Model

Protocol participants: In the model, there are two kinds of participants: mobile node MN and access point AP, which have unique identities *ID_MN_* and *ID_AP_*, respectively. Each instance of a participant (*U* or *V*) is molded as an oracle, denoted by $\Pi_{MN}^{n}$ ( $\Pi_{AP}^{n}$, respectively), meaning the *n*-th running instance of the participant MN (AP, respectively).

Protocol execution: In the model, it is assumed that an adversary 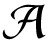 fully controls over the communication channels and can create several concurrent instances of the protocol. The public parameters *params* and identity information are known to all participants, including the adversary. During the execution of the protocol, the interaction between the adversary and the protocol participants occurs only via oracle queries, which models the adversary capabilities in a real attack. At any time, the adversary makes the following queries:

(1) *Execute*( $\Pi_{U}^{n}$, $\Pi_{V}^{m}$): This query models passive attacks, where the attacker gets access to honest executions between instances $\Pi_{U}^{n}$ and $\Pi_{V}^{n}$ by eavesdropping. The output of this query is the complete transcript that was transported during the honest execution of the protocol.
(2) *Send*( $\Pi_{U}^{n}$, *M*): This query models an active attack against an MN or AP, in which the adversary sends a message to the instance $\Pi_{U}^{n}$. The output of this query is the message that the instance $\Pi_{U}^{n}$ generates upon receipt of the message *M*.
(3) *Reveal*( $\Pi_{U}^{n}$): This query models the misuse of session keys. Only if the session key of the instance $\Pi_{U}^{n}$ is defined, the query is available and returns to the adversary the session key.
(4) *Test*( $\Pi_{U}^{n}$): This query is to measure the semantic security of the session key of instance $\Pi_{U}^{n}$: If the session key is not defined, it returns ⊥. Otherwise, it returns either the session key held by the instance if *b* = 0 or a random number of the same size if *b* = 1, where *b* is the hidden bit previously selected at random before the protocol runs.
(5) *Corrupt*(*ID_U_*): This query models the exposure of the long-term secret key. When the adversary makes this query, the oracle returns the private key corresponding to *ID_U_*.

### 3.2. Security Definitions

Notation: An instance $\Pi_{U}^{n}$ is said to be opened if the query *Reveal*( $\Pi_{U}^{n}$) has been made by the adversary. We say an instance $\Pi_{U}^{n}$ is unopened if it is not opened. An instance $\Pi_{U}^{n}$ is said to be accepted if it goes into an accept state after receiving the last expected protocol message.

Partnering: We say two instances $\Pi_{U}^{n}$ and $\Pi_{V}^{m}$ are partners if the following conditions are met: (1) they are an MN and an AP, respectively; (2) both $\Pi_{U}^{n}$ and $\Pi_{V}^{m}$ are accepted; (3) both $\Pi_{U}^{n}$ and $\Pi_{V}^{m}$ share the same session ID *sid*; (4) the partner identification for $\Pi_{U}^{n}$ is $\Pi_{V}^{m}$ and *vice versa*; and (5) no instance other than $\Pi_{U}^{n}$ and $\Pi_{V}^{m}$ accepts with a partner identification equal to $\Pi_{U}^{n}$ or $\Pi_{V}^{m}$.

Freshness: If an instance $\Pi_{U}^{n}$ has been accepted, both the instance and its partner are unopened and they are both instances of honest clients, we say the instance $\Pi_{U}^{n}$ is fresh.

Semantic security: The security notion is defined in the context of executing an ID-based handover authentication protocol *P* in the presence of an adversary 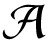. During the protocol execution, 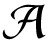 is allowed to make multiple *Execute*, *S end* and *Reveal* queries, but at most, one *Test* query, to a fresh instance of an honest participant. Finally 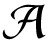 outputs a bit guess *b′* for the bit *b* hidden in the Testoracle. The adversary 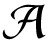 is said to be successful if *b′* = *b*. We denote the event by *S ucc* and define the advantage of 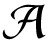 in violating the semantic security of the protocol *P* as follows:

$${\text{Adv}}_{A,P}(k,t)=2\cdot \text{Pr}[\text{S ucc}]-1$$

where *k* is the security parameter and *t* is the time parameter.

We say a handover authentication protocol *P* is semantically secure if the advantage ${\text{Adv}}_{A,P}(k,t)$ is negligible.

Authentication security: To measure the security of a handover authentication protocol resisting the impersonation attacks, we consider the mutual authentication between MN and AP. We denote by ${\text{Auth}}_{A,P}^{MN\to AP}(k,t)$ (or ${\text{Auth}}_{A,P}^{AP\to MN}(k,t)$, respectively) the probability that an adversary 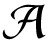 successfully impersonates an MN instance during executing the protocol *P*, while the target AP (or MN, respectively) does not detect it, where *k* is the security parameter and *t* is the time parameter. We say a handover authentication protocol *P* is mutual authentication secure if both ${\text{Auth}}_{A,P}^{MN\to AP}(k,t)$ and ${\text{Auth}}_{A,P}^{AP\to MN}(k,t)$ are negligible in the security parameter.

## 4. Review of the Protocol

In this section, we review He *et al.*'s improved protocol [15], which is very similar to the original PairHand and consists of four phases: system initialization, handover authentication, batch authentication and denial-of-service (DoS) attack resistance. The only differences between the two versions appear at the selection of the group order in the system initialization phase and the computation of the hash value of the authentication message in the handover authentication phase, and our attack is exactly to address these two phases. Below, we only review the first two phases of the improved PairHand protocol. For more details, please refer to [14].

### 4.1. System Initialization

The AS randomly chooses a value *s* ∈ ${\mathbb{Z}}_{q}^{*}$ as the master key and a generator *P* of 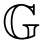, computes the corresponding public key *P_pub_* = *sP* and selects two cryptographic hash functions *H*_1_ and *H*_2_, where *H*_1_ : { 0, 1}∗ → 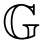 and $H_{2}:\{0,1\}*\to {\mathbb{Z}}_{q}^{*}$. The resulting public system parameter, *params*, is { 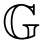, 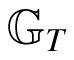, *q*, *P*, *P_pub_*, *H*_1_, *H*_2_}, and the private secret of AS is *s*. For each AP, AS computes *H*_1_(*ID_AP_*) and *sH*_1_(*ID_AP_*) as the public and private keys of that AP, respectively, and delivers them to the AP via a secure channel, where *ID_AP_* is the identity of the AP.

For the registration of a qualified MN *i* with real identity *ID_i_*, AS generates a family of unlinkable pseudo-IDs *PID* = {*pid*_1_, *pid*_2_, ⋯}, computes the public key *H*_1_(*pid_j_*) and the corresponding private key *s* · *H*_1_(*pid_j_*) for each pseudo-ID *pid_j_* ∈ *PID* and, finally, securely sends to MN *i* all tuples (*pid_j_*, *sH*_1_(*pid_j_*)). The use of shorter-lived pseudonyms is to protect each MN's privacy, preventing them from being traced.

### 4.2. Handover Authentication

**Figure 1.**
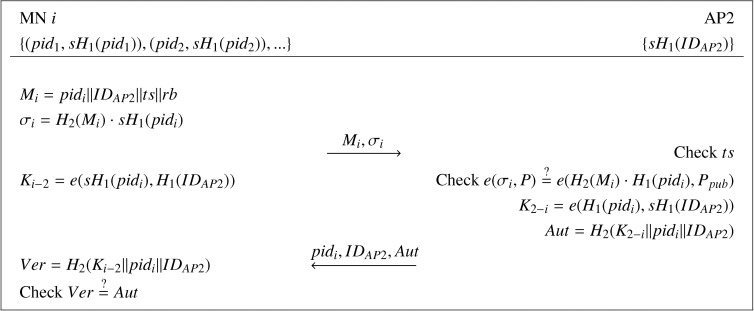
The handover authentication phase in He *et al.*'s improved PairHand protocol.

When an MN, say *i*, moves into the communication range of a new AP (AP2), a handover authentication process, which is shown in Figure 1, will be performed between MN *i* and AP2 in the following steps.

(1) MN *i* firstly picks an unused pseudo-ID *pid_i_* from his pseudo-ID family and the corresponding private key *sH*_1_(*pid_i_*). Then, MN *i* generates an authentication message as *M_i_* = *pid_i_*‖*ID_AP_*_2_‖*ts*, where *ts* is a timestamp, which is used to resist against replay attacks and “‖” denotes the concatenation of messages and checks whether *H*_2_(*M_i_*) and *q* are co-prime or not. If *H*_2_(*M_i_*) and *q* are not co-prime, it does nothing; otherwise, it continues to append redundant bits *rb* into *M_i_* until *H*_2_(*M_i_*) and *q* are not co-prime. After that, MN *i* computes the signature *σ_i_* = *H*_2_(*M_i_*) · *sH*_1_(*pid_i_*) and unicasts the access request message {*M_i_*, *σ_i_*} to AP2. Finally, MN *i* computes the session key with AP2 as *K_i_*_−_*_2_* = *e*(*sH*_1_(*pid_i_*), *H*_1_(*ID_AP_*_2_)).
(2) Upon receiving the request message {*M_i_*, *σ_i_*}, AP2 firstly checks whether the timestamp *ts* is valid. If invalid, the request is rejected. Otherwise, AP2 verifies if *e*(*σ_i_*, *P*) = *e*(*H*_2_(*M_i_*) · *H*_1_ (*ID_pid_i__*), *P_pub_*). If true, AP2 computes the session key *K*_2−_*_i_* = *e*(*H*_1_(*pid_i_*), *sH*_1_(*ID_AP_*_2_)) and the authentication code *Aut* = *H*_2_(*K*_2−_*_i_*‖*pid_i_*‖*ID_AP_*_2_) and, then, sends the tuple {*pid_i_*, *ID_AP_*_2_, *Aut*} to MN *i*.
(3) Upon receipt of the message {*pid_i_*, *ID_AP_*_2_, *Aut*}, MN *i* computes the verification code *Ver* = *H*_2_(*K_i_*_−2_‖*pid_i_*‖*ID_AP_*_2_) and compares it with *Aut*. If they are equal, MN *i* confirms that AP2 is legitimate, and the generated session key is valid. Otherwise, MN *i* cancels the connection.
(4) At last, AP2 securely transports {*M_i_*, *σ_i_*} to AS. By receiving this message, AS can identify the real identity of MN *i* according to the pseudo-ID in *M_i_*.

After successfully executing the handover protocol, MN *i* and AP2 share a session key, since *K_i_*_−2_ = *e*(*sH*_1_(*pid_i_*)),*H*_1_(*ID_AP_*_2_)) = *e*(*H*_1_(*pid_i_*),*H*_1_(*ID_AP_*_2_))*^s^* = *e*(*H*_1_(*pid_i_*),*sH*_1_(*ID_AP_*_2_)) = *K*_2−_*_i_*. Furthermore, the use of a pseudo-ID enables unilateral anonymous authentication for the MN *i*, and each session is uniquely identified by (*pid_i_*, *ID_AP_*_2_).

## 5. Attack on the Protocol

For the original PairHand protocol, what enables an attacker to recover the private key *sH*_1_(*pid_i_*) is that when *H*_2_(*M_i_*) and *q* are co-prime, he can use the inverse *u* of *H*_2_(*M_i_*) modulo *q* to get *u*· *σ_i_* = *u* · *H*_2_(*M_i_*) · *sH*_1_(*pid_i_*) = *sH*_1_(*pid_i_*)(mod*q*), since *u* · *H*_2_(*M_i_*) = 1(mod*q*). The countermeasures of He *et al.* [15] are to restrict the group order *q* to be a composite and to append redundant bits into the request message *M_i_* to ensure that the resulting *H*_2_(*M_i_*) and *q* are not co-prime. By doing this, it seems that the private key *sH*_1_(*pid_i_*) will not be exposed by the signature *σ_i_*, since there is no modular inverse for *H*_2_(*M_i_*).

However, the following attack will show that He *et al.*'s improved protocol [15] does not eradicate the design weakness. Our attack is based on the assumption [6] that adversary 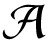 has total control over all communication channels between AP2 and MN *i*. This means that the adversary may intercept, delete or modify any message in the channels. Suppose that MN *i* requests the service of a new access point AP2 by sending the message (*M_i_*, *σ*) (where *M_i_* = *pid_i_*‖*ID_AP_*_2_‖*ts*‖*rb* and *σ_i_* = *H*_2_(*M_i_*) · *sH*_1_(*pid_i_*)) in a wireless channel, which is dominated by the adversary 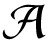. 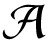 interrupts the request message, so that MN *i* will not receive the response from AP2. After a certain delay, MN *i* will regenerate a new request message $\left(M_{i}^{\prime },\sigma_{i}^{\prime }\right)$ and send it to AP2, where $M_{i}^{\prime }={\text{pid}}_{i}\left\|ID_{AP2}\right\|ts^{\prime }\|rb^{\prime }$, $\sigma_{i}^{\prime }=H2(M_{i}^{\prime })\cdot sH_{1}({\text{pid}}_{i})$, *ts′* denotes a new timestamp and *rb′* is a new redundant bit string. Since 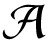 controls the whole network communication, it can easily capture the new message. Once the adversary 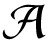 owns two authentication messages (*M_i_*, *σ*) and $\left(M_{i}^{\prime },\sigma_{i}^{\prime }\right)$ corresponding to the same pseudo-ID *pid_i_*, it randomly selects two values *x*_1_, *x*_2_ ∈ ℤ*_q_* and computes:

$$\begin{array}{ll} \alpha & =(x_{1}\cdot \sigma_{i})+(x_{2}\cdot \sigma_{i}^{\prime })\\ & =(x_{1}\cdot H_{2}(M_{i})sH_{1}({\text{pid}}_{i}))+(x_{2}\cdot H_{2}(M_{i}^{\prime })sH_{1}({\text{pid}}_{i}))\\ & =(x_{1}\cdot H_{2}(M_{i})+x_{2}\cdot H_{2}(M_{i}^{\prime }))sH_{1}({\text{pid}}_{i}). \end{array}$$

Then, 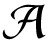 directly computes $\beta =(x_{1}\cdot H_{2}(M_{i})+x_{2}\cdot H_{2}(M_{i}^{\prime }))(\mathrm{mod}q)$ by using *M_i_* and $M_{i}^{\prime }$ and checks whether *β* and *q* are co-prime. If yes, it generates $\gamma ={\left(x_{1}\cdot H_{2}(M_{i})+x_{2}\cdot H_{2}(M_{i}^{\prime })\right)}^{-1}(\mathrm{mod}q)$ and computes the private key *sH*_1_(*pid_i_*) = γ · *α*. Otherwise, it reselects random values *x*_1_, *x*_2_ and computes *α* and *β* again, until *β* and *q* are co-prime.

As *q* is a randomly generated system parameter, *M_i_* and $M_{i}^{\prime }$ are random messages and *x*_1_ and *x*_2_ are randomly chosen from ℤ*_p_*, we can approximately view *q* and $\beta =(x_{1}\cdot H_{2}(M_{i})+x_{2}\cdot H_{2}(M_{i}^{\prime }))(\mathrm{mod}q)$ as two independent random numbers. Let {*p*_1_, *p*_2_, *p*_3_, ⋯} denote the ascending sequence of all prime numbers, and let *p_k_* be the largest prime number less than *q*. The probability that *β* is divisible by a prime *p_i_* is $\frac{1}{p_{i}}$, where *i* ≤ *k*, and the same fact holds for *q*. Therefore, the probability that the two numbers *β* and *q* are both divisible by this prime number is $\frac{1}{p_{i}^{2}}$, whilst the probability that at least one of them is not is $1-\frac{1}{p_{i}^{2}}$. Thus, the probability of the success of our attack for one time, *P_success_*, which is equal to the probability that *β* and *q* are co-prime, is:

$$\begin{array}{ll} P_{\text{success}} & =\prod_{i=1}^{k}\left(1-\frac{1}{p_{i}^{2}}\right)\\ & >\prod_{i=1}^{\infty }\left(1-\frac{1}{p_{i}^{2}}\right)\\ & =\prod_{i=1}^{\infty }{\left(1-\frac{1}{p_{i}^{-2}}\right)}^{-1}\\ & =\frac{6}{\pi^{2}}\\ & \approx 0.6079. \end{array}$$

Obviously, a natural way to cope with the above attack is to ensure that each pseudo-ID is used only one time, regardless of whether the AP responds correctly, which will require different signatures to correspond to different pseudo-IDs. As a consequence, it is impossible for an adversary to compute out the private key by using linear combinations of two signatures. However, this countermeasure will largely reduce the availability of the handover authentication protocol and give rise to more serious security problems, as shown as follows. When an MN moves and leaves from the service range of its old AP, it will attempt to connect to and identify a new AP by instantly sending authentication messages. Once a pseudo-ID is used only one time, an attempt to connect will cost one pseudo-ID of the MN, which will cause a great waste on the pseudo-IDs and force the MN to store a much larger number of pseudo-IDs. However, mobile nodes are often lightweight devices and have limited storage spaces, this makes them unable to afford a large number of redundant pseudo-IDs. Additionally, the increase of the number of pseudo-IDs will lead to the growth of the length of pseudo-IDs, which deeply affects the efficiency of the computation and the communication of the whole wireless network. More seriously, if there is an adversary who always interrupts the request authentication message of an MN, the MN will eventually use up all its pseudo-IDs and be out of the service of the system, due to instantly repeating the request. Such an attack can be avoided by using additional precautions, such as delaying the response or introducing exponentially increasing delays after failed attempts and switching to other AP after an excessive amount of failures. However, all of these measures are very costly and can cause more additional risks, which is contrary to the design rational of PairHand.

## 6. Our Handover Authentication Protocol

According to the above analysis, the point to overcome the security weakness of the two PairHand protocols is to provide a secure authentication mechanism for the first message transmission. Below, we provide a simple scheme, which not only eliminates the security risks mentioned above, but greatly preserves the desirable efficiency features of the original protocol. Similar to PairHand, the proposed scheme is composed of four phases: system initialization, handover authentication, batch authentication and DoS attack resistance, where the first phase and the fourth phase are the same as those of the PairHand protocol. For the sake of completeness, all of the four phases are fully described in the following.

### 6.1. System Initialization

Let 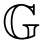 be a cyclic additive group of composite order *q* and 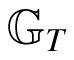 be a cyclic multiplicative group of the same order. Let *P* be a generator of 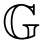 and *e* be a bilinear map *e* : 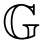 × 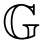 → 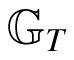.

The AS randomly chooses a value $s\in {\mathbb{Z}}_{q}^{*}$ as the master key, computes the corresponding public key *P_pub_* = *sP* and selects two cryptographic hash functions *H*_1_ and *H*_2_, where *H*_1_ : {0, 1}∗ → 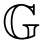 and $H_{2}:\{0,1\}*\to {\mathbb{Z}}_{q}^{*}$. The resulting public system parameter, *params*, is { 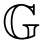, 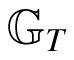, *q*, *P*, *P_pub_*, *H*_1_, *H*_2_}, and the private secret of AS is *s*. For each AP, AS computes *H*_1_(*ID_AP_*) and *sH*_1_(*ID_AP_*) as the public and private keys of that AP, respectively, and delivers them to the AP via a secure channel, where *ID_AP_* is the identity of the AP.

For the registration of a qualified MN *i* with real identity *ID_i_*, AS generates a family of unlinkable pseudo-IDs *PID* = {*pid*_1_, *pid*_2_, ⋯}, computes the public key *H*_1_(*pid_j_*) and the corresponding private key *s* · *H*_1_(*pid_j_*) for each pseudo-ID *pid_j_* ∈ *PID* and, finally, securely sends to MN *i* all tuples (*pid_j_*, *sH*_1_(*pid_j_*)).

### 6.2. Handover Authentication

When an MN, say *i*, moves into the communication range of a new AP (AP2), a handover authentication process will be performed between MN *i* and AP2 in the following steps.

(1) MN *i* firstly picks an unused pseudo-ID *pid_i_* and the corresponding private key *sH*_1_(*pid_i_*) and computes *M_i_* = (*pid_i_*‖*ID_AP_*_2_‖*ts*), where *ts* is the timestamp. Then, MN *i* chooses a random value $r_{i}\in {\mathbb{Z}}_{q}^{*}$, which is a nonce, computes *R_i_* = *r_i_P* and *σ_i_* = *H*_2_(*M_i_*‖*R_i_*) · *sH*_1_(*pid_i_*) + *r_i_P_pub_* and unicasts the access request message *M_i_* and its signature pair (*R_i_*, *σ_i_*) to AP2. Finally, it computes the session key with AP2 as *K_i_*_−2_ = *e*(*sH*_1_(*pid_i_*), *H*_1_(*ID_AP_*_2_)).
(2) Upon receiving the message {*M_i_*, *r_i_*, *σ_i_*}, AP2 checks the timestamp *ts*. If invalid, the request is rejected. Otherwise, AP2 verifies if *e*(*σ_i_*, *P*)=*e*(*H*_2_(*M_i_*‖*R_i_*)*H*_1_(*pid_i_*) + *R_i_*, *P_pub_*). If true, AP2 computes the session key *K*_2−_*_i_* = *e*(*H*_1_(*pid_i_*), *sH*_1_(*ID_AP_*_2_)) and the authentication code *Aut* = *H*_2_(*K*_2−_*_i_*‖*pid_i_*‖*ID_AP_*_2_) and, then, sends the tuple {*pid_i_*, *ID_AP_*_2_, *Aut*} to MN *i*.
(3) Upon receipt of the message {*pid_i_*, *ID_AP_*_2_, *Aut*}, MN *i* computes the verification code *Ver* = *H*_2_(*K_i_*_−2_‖*pid_i_*‖*ID_AP_*_2_) and compares it with *Aut*. If they are equal, MN *i* confirms that AP2 is legitimate and that the generated session key is valid. Otherwise, MN *i* cancels the connection.
(4) At last, AP2 securely transports {*M_i_*, *σ_i_*} to AS. By receiving this message, AS can identify the real identity of MN *i* according to the pseudo-ID in *M_i_*.

**Figure 2.**
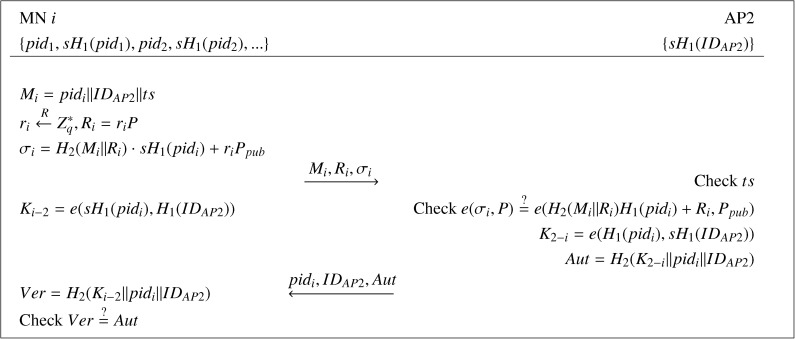
The handover authentication phase in our protocol.

The handover authentication phase of the proposed scheme is also shown in Figure 2.

### 6.3. Batch Authentication

A mass of signature verifications is likely to cause the potential bottleneck at APs. Batch authentication [14] is a desirable feature to solve the problem, which allows APs to verify multiple signatures simultaneously. Its advantage lies in that the total computation cost in the verification performed by APs can be apparently reduced.

Our protocol still enjoys the batch authentication feature. Suppose *n* request messages {*M*_1_, *R*_1_, *σ*_1_}, {*M*_2_, *R*_2_, *σ*_2_}, ⋯, {*M_n_*, *R_n_*, *σ_n_*}, come simultaneously from *n* distinct MNs, MN 1, MN 2, ⋯, MN *n*, respectively The target AP can perform a batch verification on these *n* signatures as follows:

$$\begin{array}{ll} & e(\sum_{i=1}^{n}\sigma_{i,}P)\\ = & e(\sum_{i=1}^{n}(H_{2}(M_{i}∥r_{i})\cdot sH_{1}({\text{pid}}_{i})+r_{i}P_{\text{pub}}),P)\\ = & e(\sum_{i=1}^{n}(H_{2}(M_{i}∥r_{i})\cdot sH_{1}({\text{pid}}_{i})+r_{i}sP),P)\\ = & e(\sum_{i=1}^{n}(H_{2}(M_{i}∥r_{i})\cdot H_{1}({\text{pid}}_{i})+R_{i}),P_{\text{pub}}) \end{array}$$

From the above equation, it is obvious that the computation cost of verifying *n* signatures is dramatically reduced to *n* point multiplication and two pairing operations by using the batch processing.

### 6.4. DoS Attack Resistance

In the handover authentication circumstance, DoS attack is an attempt to exhaust the resources of AP and AS and make them unavailable to its intended partners. A usual manner adopted by the adversary is to inject bogus access requests to the networks, forcing the APs to perform expensive cryptographic verifications and eventually exhaust their resources.

The proposed scheme still adopts the polynomial-based lightweight verification of PairHand [14] to resist the DoS attack. In the system initialization phase, AS randomly generates a bivariate *t*-degree polynomial $f(x,y)=\sum_{i,j=0}^{t}a_{ij}x^{i}y^{j}$ over a prime field *F_p_*, such that *f*(*x*, *y*) = *f*(*y*, *x*). When MN *i* registers to AS, for each pseudo-ID *pid_i_*, AS computes *f*(*pid_i_*, *y*), which is a polynomial share of *f*(*x*, *y*), and then securely transmits them to MN *i*. Furthermore, AS computes and deliveries *f*(*ID_AP_*, *y*) to each AP, where *ID_AP_* is the identity of the AP. As the evaluation of the polynomial is very fast [14], each AP can perform a lightweight verification on the access request from MN *i* by checking *f*(*pid_i_*, *ID_AP_*) ≟ *f*(*ID_AP_*, *pid_i_*), where the former is computed by MN *i* with *f*(*pid_i_*, *y*) at point *ID_AP_* and the later is done by the AP with *f*(*ID_AP_*, *y*) at point *pid_i_*. Once an AP is under attack, it starts the above measure, adding “Yes” and its identity into the beacon messages. As a result, DoS attack can be effectively mitigated, since each AP can promptly verify the authorized user with the communication key before conducting expensive cryptographic verifications.

### 6.5. Security Analysis

#### Theorem 1

*Assume hash functions H*_1_ *and H*_2_ *are random oracles. Let* 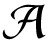 *be a probabilistic polynomial time Turing machine. Let Q_s_, Q*_1_ *and Q*_2_ *respectively denote the number of queries that* 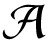 *can ask of the Sendoracle, the number of queries that* 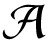 *can ask of the H*_1_ *random oracle and the number of queries that* 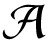 *can ask of the H*_2_ *random oracle. If the attacker* 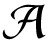 *can successfully violate the MN-to-AP authentication security of the protocol within time T, with probability ε* ≥ 10(*Q_s_* + 1)(*Q_s_* + *Q*_2_)/*q, then another probabilistic polynomial time Turing machine* 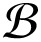 *can be built to utilize* 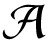 *to break the CDH problem in expected time* $T^{\prime }\leq 120686Q_{1}^{2}Q_{2}T/ɛ$.

#### Proof

Suppose that the attacker 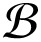 is given a challenging CDH triple (*P*, *aP*, *sP*) and its goal is to compute *asP*. 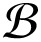 runs 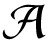 as a subroutine and simulates the environment for attacking the protocol. According to the challenging instance, 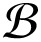 provides 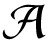 the public parameters ( 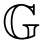, 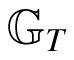, *e*, *q*, *P*, *P_pub_*, *H*_1_, *H*_2_), such that *P_pub_* = *sP*.

Without loss of generality, we assume that for any pseudo-ID, the adversary 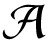 invokes *H*_1_, *H*_2_, *S end*, *Execute* and *Corrupt* at most once. To provide consistent responses for these queries, 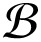 maintains two lists *L*_*H*_1__ and *L*_*H*_2__, which are initially empty.

**H_1_**-query: When 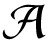 invokes an *H*_1_ query for *pid_i_*, 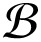 checks whether *pid_i_* = *pid_U_*. If yes, 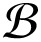 returns *aP*. Otherwise, 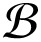 returns a randomly selected value *h* ∈ 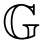 and appends < *pid_i_*, *h* > into the list *L*_H_1__.
**H_2_**-query: When 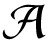 invokes an *H*_2_ query for messages (*m*, *R*), 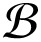 returns a random number $t\in {\mathbb{Z}}_{q}^{*}$ and stores < *m*, *R*, *t* >.
**Corrupt**-query: If the queried pseudo-ID is legal and is not equal to *pid_U_*, 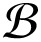 searches the corresponding item in the list *L_H_*_1_ according to the pseudo-ID and then returns the secret key. Otherwise, 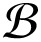 returns ⊥.
**Execute**-query: This query is responded to by invoking the corresponding *S end* queries.
**Send**-query: When 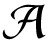 invokes a send $\left(\Pi_{i}^{n},m\right)$ query, simulator 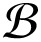 extracts *pid_i_* involved in the query and uses it to invoke query *H*_1_. Then, 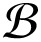 randomly chooses *r_i_*, $t\in {\mathbb{Z}}_{q}^{*}$, computes *σ_i_* = *r_i_P_pub_*, *R_i_* = *r_i_P* − *tH*_1_(*pid_i_*) and stores the item < *m*, *R_i_*, *t* > in the list *L_H_*_2_. Finally, it outputs (*pid_i_*, *R_i_*, *σ_i_*). If there is no collision of queries to the random oracle during the process, 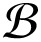 can successfully simulate the protocol environment in front of 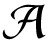, due to the fact that the probabilities of the duple (*α*, *β*, *γ*, *δ*), such that $\beta \in {\mathbb{Z}}_{q}^{*}$, *α*, *γ*, *δ* ∈ 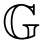 and *e*(*γ*, *P*) = *e*(*βα* + *δ*, *sP*) appear, the following two distributions Γ and Γ′ are the same.

$$\begin{array}{ccc} \Gamma^{\prime }=\left\{(h,t,\sigma ,R)|\begin{array}{c} r,t\in_{R}{\text{Z}}_{q}^{*}\\ h\in_{R}\text{G}\\ R=rP-th\\ \sigma =\text{rsP} \end{array}\right\} & \text{and} & \Gamma =\left\{(h,t,\sigma ,R)|\begin{array}{c} r,t\in_{R}{\text{Z}}_{q}^{*}\\ h\in_{R}\text{G}\\ R=rP\\ \sigma =\text{tsh}+\text{rsP} \end{array}\right\} \end{array}$$

According to the Forking lemma [21], if 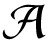 outputs a valid authentication message tuple (*pid_U_*, *m*, *σ*, *R*), after a polynomial replay of the attacker 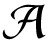 with the same tape, but different choices of *H*_2_, 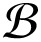 obtains two valid message tuples (*pid_U_*, *m*, *σ* = *tsaP* + *rsP*, *R* = *rP*) and (*pid_U_*, *m*, *σ′* = *t′saP* + *rsP*, *R* = *rP*) with *t* ≠ *t′* and eventually resolves the CDH challenge by computing *asP* = (*σ* − *σ′*)/(*t* − *t′*).

Additionally, the probability of that the two forged authentication messages correspond to *pid_U_* is $1/Q_{1}^{2}$. As a result, the upper bound of the expected time for breaking the CDH problem will be expanded $Q_{1}^{2}$-times the one in the Forking lemma.

#### Theorem 2

*Assume hash functions H_1_ and H_2_ are random oracles. Let* 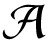 *be a probabilistic polynomial time Turing machine. If the attacker* 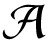 *can successfully violate the AP-to-MN authentication security of the protocol, then another probabilistic polynomial time Turing machine* 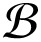 *can be built to utilize* 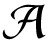 *to break the BDH problem*.

#### Proof

Let (*P*, *aP*, *bP*, *sP*) be the BDH instance provided to the simulator 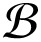. To simulate the attacking environment for 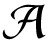, 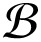 publishes the public parameters ( 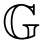, 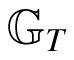, *e*, *q*, *P*, *P_pub_*, *H*_1_, *H*_2_), such that *P_pub_* = *sP* and maintains two hash lists *L_H_*_1_ and *L_H_*_2_, which are initially empty. Without loss of generality, we assume that for any pseudo-ID or AP identity, the adversary 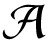 invokes *H*_1_, *H*_2_, *S end*, *Execute* and *Corrupt* at most once. Let *Q_M_* and *Q_A_* be the number of queries that 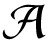 can ask of the random oracle *H*_1_ for MN nodes and the number of queries that 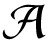 can ask of the random oracle *H*_1_ for AP nodes, respectively. 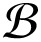 guesses the target session between the MN *pid_U_* and the AP *ID_V_*, which are randomly chosen.

**H_1_**-query: If 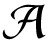 makes an *H*_1_ query for *pid_U_*, 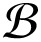 returns *aP*. If the query is for the AP identification *ID_V_*, 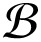 returns *bP*. Otherwise, 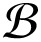 returns a randomly selected value *h* ∈ 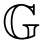 and adds < *pid_i_*, *h* > or < *ID_V_*, *h* > into the list *L_H_*_1_.
**H_2_**-query: When 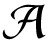 invokes an *H*_2_ query for messages *M*, 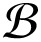 chooses a random number *t* ∈ ℤ*_q_*, stores *< M*, *t* > the list *L_H_*_2_ and then returns it.
**Corrupt**-query: If the queried identity is legal and is not equal to *pid_U_* and *ID_V_*, 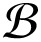 searches the corresponding item in the list *L_H_*_1_ according to the identity and then returns the secret key. Otherwise, 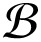 returns ⊥.
**Execute**-query: This query is responded to by invoking the corresponding *S end* queries.
**Send**-query: There are two types of *S end* queries: MN-to-AP and AP-to-MN, denoted by *S end*1 and *Send*2, respectively. 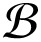 answers them by invoking the *H*_1_ and *H*_2_ queries.
  - If the query is *Send*1, simulator 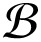 randomly chooses *r_i_*, $t\in {\mathbb{Z}}_{q}^{*}$ and computes *σ_i_* = *r_i_P_pub_*, *R_i_* = *r_i_P* − *tH*_1_(*pid_i_*) by invoking *H*_1_ queries with *ID_i_*. Then, it adds the item (*m*, *R_i_*, *t*) in the list *L_H_*_2_ and outputs message tuple (*pid_i_*, *R_i_*, *σ_i_*).
  - If the query is *S end2*, simulator 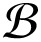 checks whether *e*(*σ_i_*, *P*)=*e*(*H*_2_(*M_i_*‖*R_i_*)*H*_1_(*pid_i_*) + *R_i_*, *P_pub_*). If false, 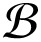 outputs “⊥”. Otherwise, 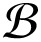 chooses a random value *k* ∈ 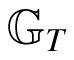 and computes *aut* = *H*_2_(*k*‖*pid_i_*‖*ID_j_*) by making the *H*_2_ query. Finally, it returns the message tuple (*aut*‖*pid_i_*‖*ID_j_*).

The success of 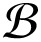 breaking the BDH problem denotes the event that *pid_U_* and *ID_V_* are partners and 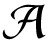 asks the *H*_2_ query with a tuple (*K*‖*pid_U_*‖*ID_V_*) where *K* = *e*(*P*, *P*)*^abs^*. According to the above simulation, the probability that *pid_U_* and *ID_V_* are partners is 1/*Q_m_Q_a_*. Therefore, if 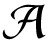 outputs a valid authentication message with probability *ε*, the probability of the success of 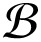 is less than *ε/Q_M_Q_A_*.

#### Theorem 3

*Assume hash functions H*_1_ *and H*_2_ *are random oracles. If the protocol enjoys the mutual authentication security, it is also semantically secure*.

#### Proof

To prove the semantic security for the protocol, we apply the same simulation way used in proving Theorem 2. Let *F*_1_ (or *F*_2_, respectively) denote the event that the attacker successfully forges an MN-to-AP (or AP-to-MN, respectively) authentication message. Let *S*_0_ (or *S*_1_, respectively) denote the event that in the real (or simulated, respectively) attacking game, the attacker successfully guess the challenge bit involved in the *Test* oracle. If both the events *F*_1_ and *F*_2_ do not happen, the real and simulated games proceed identically, and we get the following equation:

$$\text{Pr}[S_{0}\Lambda^{\neg }F_{1}\Lambda^{\neg }F_{2}]=\text{Pr}[S_{1}\Lambda^{\neg }F_{1}\Lambda^{\neg }F_{2}]\text{.}$$

On the other hand, it is obvious that in the simulation, the attacker cannot obtain any information about the protocol session key, since the session key is a randomly chosen value not related to any message transported in the public information channel. This means that the attacker can only guess the hidden bit, so that *Pr*[*S*_1_] = 1/2. Following the difference lemma [22], we get

$$\left|\text{Pr}[S_{0}]-\text{Pr}[S_{1}]\right|\leq \text{Pr}[F_{1}\vee F_{2}]\leq \text{Pr}[F_{1}]+\text{Pr}[F_{2}].$$

According to the definition of the semantic security for the protocol, then we have ${\text{Adv}}_{A,P}(k,t)\leq 2\cdot (\text{Pr}[F_{1}]+\text{Pr}[F_{2}])=2\cdot {\text{Auth}}_{A,P}^{MN\to AP}(k,t)+2\cdot {\text{Auth}}_{A,P}^{AP\to MN}(k,t)$, □

### 6.6. Performance Comparison

Compared with the existing handover authentication protocols, the proposed protocol has the advantage in communication, computation and security. For those protocols prior to PairHand [1–4,6–10,12], its superiority comes through the low burden on AS, the two-run handshakes between MN and AP, the batch authentication and the privacy protection for MN. To evaluate its advantage over the post-PairHand protocols [14,15,17], we mainly consider its performance superiority on secret key size, computational cost and security features. In Table 1, we present the comparison results on these aspects among He *et al.*'s improved PairHand [15], Tsai *et al.*'s [17] protocol and our scheme. For computational cost, we focus on the time spent on the high cost operations, such as the time spent on the paring operations (*T_p_*), the time spent on the multiplications on the elliptic curve (*T_m_*) and the time spent on the search for non-co-primes (*T_s_*), while the time spent on highly efficient operations, such as the hash function and the scalar addition on the elliptic curve, is neglected. The estimate of the time consumption at an MN is based on He *et al.*'s work in [14,15], where by using the MNT curve with the order of 160 bits and the degree *k* = *6* and the MIRACL and PBC libraries (c/c++), an MN runs on an 800 MHz processor. To evaluate the length of the messages transmitted in the protocol execution, we assume that the lengths of *pid_i_*, *ts* and *ID_AP_*_2_ are four, two and four bytes, respectively. We notice that the computational time of our protocol and Tsai *et al.*'s protocol are much lower than He *et al.*'s protocol, due to their prime-order work groups. This is because the composite order in He *et al.*'s protocol should be at least 1024-bit to be infeasible to factorize, while a 160-bit prime order is enough to achieve the same security level. An estimation [23] shows that the composite-order pairing is roughly 50-times slower than its prime-order counterpart. For security, both our scheme and Tsai *et al.*'s protocol enjoy provable security, but He *et al.*'s protocol does not. In terms of the secret key size, our protocol is superior to Tsai *et al.*'s protocol and is the same as the original PairHand protocol [14]. As a result, our scheme can be easily implanted to the running environment of the original PairHand protocol without any change to the public and private parameters.

## 7. Conclusions

In this paper, in reviewing the PairHand family protocols, we present a stronger key recovery attack on an improved PairHand protocol, which requires less signatures to be generated with the same private key compared with the existing attacks. Consequently, we present a new handover authentication protocol and prove its security in the random oracle model. Compared with the two latest handover authentication protocols, the proposed protocol has the advantages of efficiency and security.

## Figures and Tables

**Table 1.** Protocol comparisons. MN, mobile node; AP, access point.

	**He** *et al*. [15]	**Tsai** *et al*. [17]	**Ours**
The number of private keys	1	1	1
The number of Public keys	1	2	1
Provably secure	No	Yes	Yes
MN Anonymity	Yes	Yes	Yes
MN unlinkability	Yes	Yes	Yes
MN computational cost	1*T_p_* + 1*T_m_* + 1*T_s_*	1*T_p_* + 1*T_m_*	1*T_p_* + 1*T_m_*
The computation time consumption at an MN	≈ 299.332 ms	≈ 7.564 ms	≈ 7.564 ms
AP computational cost	1*T_p_*	1*T_p_*	1*T_p_*
The length of the transmitted messages	166 bytes	78 bytes	78 bytes
Work group	composite order	prime order	prime order
